# Impairment of reproductive capabilities in three subsequent generations of asymmetric hybrids between *Eisenia andrei* and *E*. *fetida* from French, Hungarian and Polish laboratory colonies

**DOI:** 10.1371/journal.pone.0235789

**Published:** 2020-07-09

**Authors:** Barbara Plytycz, Janusz Bigaj, Aleksandra Rysiewska, Artur Osikowski, Sebastian Hofman, Agnieszka Podolak, Pawel Grzmil

**Affiliations:** 1 Department of Evolutionary Immunology, Institute of Zoology and Biomedical Research, Jagiellonian University, Krakow, Poland; 2 Department of Malacology, Institute of Zoology and Biomedical Research, Jagiellonian University, Krakow, Poland; 3 Department of Animal Reproduction, Anatomy and Genomics, University of Agriculture in Krakow, Krakow, Poland; 4 Department of Comparative Anatomy, Institute of Zoology and Biomedical Research, Jagiellonian University, Krakow, Poland; 5 Department of the Basis of Agriculture and Waste Management, Institute of Agricultural Sciences, Land Management and Environmental Protection, College of Natural Sciences, University of Rzeszow, Rzeszow, Poland; 6 Department of Genetics and Evolutionism, Institute of Zoology and Biomedical Research, Jagiellonian University, Krakow, Poland; Laboratoire de Biologie du Développement de Villefranche-sur-Mer, FRANCE

## Abstract

*Eisenia andrei* (Ea) and *E*. *fetida* (Ef) lumbricid earthworms are simultaneous hermaphrodites potentially capable of self-fertilization and hybridization. We have shown previously that reproductive isolation in these species is incomplete in Ea and Ef earthworms of French provenance, as viable offspring appeared in inter-specific pairs. Fertile asymmetric hybrids developed from Ea-derived ova fertilized by Ef-derived spermatozoa, as well as Ea or Ef specimens derived after self-fertilization (resulting from admixture of endogenously produced spermatozoa with sperm from a partner), but never Ef-hybrids from Ef-ova fertilized by Ea-spermatozoa. The latter appeared only in backcrosses of Ea-hybrids with the Ef. Here we show that these phenomena are not unique for French Ea/Ef earthworms, but are shared by earthworms from French, Hungarian, and Polish laboratory cultures. Semi-quantitative studies on fertility of Ea-derived hybrids revealed gradually decreasing numbers of offspring in three successive generations, more rapid in backcrosses with Ef than with Ea, and the absence of progeny in pairs of hybrids, despite the presence of cocoons in almost all pairs. Based on species specific mitochondrial and nuclear DNA sequences, we provide the first examples of two unique sterile hybrids with mitonuclear mismatch and potential mitonuclear incompatibility among offspring of one of the hybrid+Ef pairs. Earthworms from the investigated populations did not reproduce when kept from hatching in isolation or with representatives of *Dendrobaena veneta* but started reproducing upon recognition of a related partner, such as Ea, Ef or their hybrids. The existence of Ea or Ef specimens among offspring of hybrid+Ea/Ef pairs might be explained either by partner-induced self-fertilization of Ea/Ef or hybrid-derived ova, or by cross-fertilization of Ea/Ef /hybrid ova by partner-derived spermatozoa; the latter might contribute to interspecific gene introgression.

## Introduction

The eco-physiologically similar hermaphroditic earthworms, ‘red worms’ *Eisenia andrei* (Ea) and ‘tiger worms’ (or ‘brandlings’) *Eisenia fetida* (Ef), were originally considered as pigmentation morphs of *E*. *fetida*, and later as subspecies. Currently they are treated as two distinct species, Ea and Ef, with species-specific mitochondrial and nuclear DNA sequences [1–3], the latter with Ef1 and Ef2 mitochondrial lineages [4]. Studies on the breeding biology of Ea and Ef specimens from Spain and Brazil have indicated that they are reproductively isolated [5], although the isolation is incomplete [6, 7]. Relicts of past hybridization have been recognized by species-specific DNA sequences in specimens from natural populations of Ea and Ef from Scandinavia [8]. Moreover, a growing body of evidence has revealed the existence of asymmetric hybridization between Ea and Ef1/Ef2 from French laboratory stocks with the appearance of fertile Ea-derived hybrids that may be accompanied by partner-induced self-fertilization [9–11]. On the other hand, the French Ea and Ef specimens do not reproduce when kept in isolation from a hatchling stage [11], whereas virgin Spanish earthworms of these species are capable of uniparental reproduction [12]. These apparently contradictory observations suggest the existence of various modes of reproductive isolation operating in geographically distant earthworms within *Eisenia* sp. Therefore, further studies on the breeding biology of these hermaphroditic species capable of self-fertilization are pertinent.

The aim of the present laboratory investigation was to test whether asymmetrical hybridization between Ea and Ef earthworms, partner-induced self-fertilization, and lack of reproduction in virgin isolated earthworms are unique features for earthworms of French provenance, or if they are more general phenomena. To answer these questions we investigated earthworms from French, Hungarian, and Polish laboratory stocks and 1) looked for signs of reproduction of virgin specimens kept in isolation; 2) joined adult virgin earthworms for 3–4 months in interspecific Ea+Ef pairs and genotyped their offspring; 3) repeated the same procedures with the hybrids (H) of the first, second and third generations either backcrossed with Ea or Ef or joined with other hybrids. We found that virgin earthworms did not reproduce, hybridization and facilitated self-fertilization occurred in earthworms from all tested populations, and the fertility of second and third generation hybrids was significantly or completely impaired.

## Material and methods

### Earthworms

Composting earthworms *Eisenia andrei* (Ea) and *E*. *fetida* (Ef) from French (Lille University; L), Hungarian (Pecs University; P), and Polish (Kluczbork; K) laboratory stocks, and commercially available *Dendrobaena veneta* (Dv) from Kepa Slupska (Poland) were cultured for a decade in Jagiellonian University (Krakow, Poland). Descendants of earthworms genotyped previously by mitochondrial COI genes as EaL, Ef1L, Ef2L, EaP, EfK, and Dv [9, 13–15] were reared in separate boxes and their progeny was used for the present study. Additionally, individually kept inter-specific, first generation Ea-derived hybrids from EaL+Ef2L pairs from parallel investigations [11] were also incorporated. Laboratory conditions and feeding were the same as described previously [9].

### Earthworm genotyping

Supravitally amputated tail tips of numerically coded adult earthworms were used for DNA extraction and genetic analysis using oligonucleotide primers amplifying species-specific variants of mitochondrial COI genes and nuclear 28S rRNA genes, as described earlier [9]. Sequences obtained in our previous study [11] were also used (GenBank accession numbers: MN719756-MN719757, MN719764-MN719765, MN719768-MN719769, MN719778-MN719781, MN719786-MN719793, MN719796-MN719799, MN719802-MN719807, MN719810-MN719811, MN719818-MN719821, MN719828-MN719829, MN719832-MN719833, MN719840-MN719841).

**Fig 1:**
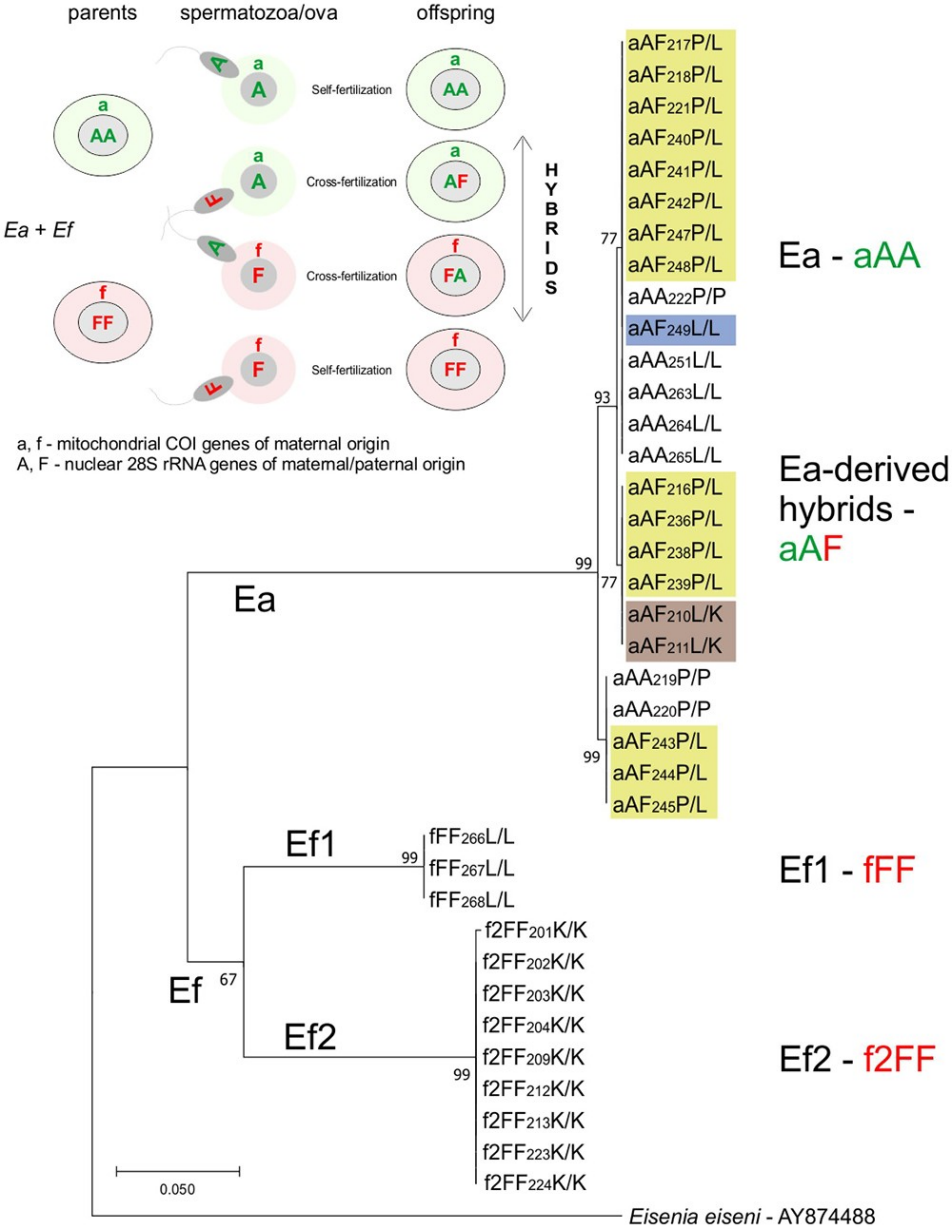
Phylogram of genotyped parents and first generation offspring. Interspecific Ea+Ef pairs of earthworms from Lille (L, France), Pecs (P, Hungary), and Kluczbork (K, Poland) (compare with Table 2) and their offspring were genotyped and the maximum-likelihood phylogram is given, considering the ‘a’ or ‘f’/’f2’ COI gene, with additional information of the ‘A’ or ‘F’ nuclear 28S rRNA genotypes of the same individuals with the same code, and L/L, P/L, or L/K symbols of the origin of parental specimens. aAA indicates Ea, fFF/f2FF means Ef1/Ef2, and aAF—hybrids. All sequences are deposited in GenBank. Inset: Scheme of inter-specific mating of Ea (aAA) and Ef (fFF) earthworms for detection of self-fertilization and hybridization. Ova of each species (aA or fF) may be fertilized either by the A or F spermatozoa of the same specimen or spermatozoa coming from the partner, giving self-fertilized pure species aAA or fFF earthworms and/or cross-fertilized aAF/fFA hybrids.

As previously established, mitochondrial species-specific sequences of the COI gene were called either ‘a’ or ‘f’/’f2’ for Ea and Ef1/Ef2, respectively, while the diploid species-specific nuclear markers of the 28S rRNA gene were called either ‘A’ or ‘F’ for Ea and Ef, respectively. The investigated specimens were genotyped either as pure species, Ea (aAA), Ef1 (fFF), Ef2 (f2FF), or inter-specific hybrids, either Ea-derived aAF, Ef-derived fFA or hybrid-derived aFF with the two first letters (aA-, fF-, aF-) representing ova-derived genes, i.e. maternity, and the last letter spermatozoa-derived genes, i.e. paternity (see inset of Fig 1).

### Experimental scheme

#### 1. Isolated virgin earthworms versus Ea+Ef pairs

To avoid uncontrolled copulations and sperm exchange, most freshly hatched earthworms, both pure species and hybrids, were kept individually in separate boxes for at least 14 weeks, and then soil in the boxes was checked for the presence of cocoons and/or hatchlings. Virgin adult earthworms were joined in Ea+Ef pairs and their reproductive activities were checked by cocoon production and hatchability.

#### 2. Inter-generic versus inter-specific pairs

In order to test whether the presence of a partner from a different species can stimulate reproductive behavior, newly hatched Ea and Ef1 were kept for 14 weeks with newly hatched specimens of *Dendrobaena veneta* (Dv) forming 6 inter-generic groups, 3 (Ea+Dv) and 3 (Ef1+Dv). Thereafter, the earthworms were rejoined for 3 months with partners from the same genus forming in new boxes 3 inter-specific Ea+Ef and 3 conspecific Dv+Dv groups. Soil in each box was checked for the presence of cocoons and hatchlings.

#### 3. Progeny of inter-specific Ea+Ef pairs

Freshly hatched earthworms were isolated for at least 14 weeks, and afterwards, adult virgin specimens were joined for 3–4 months in inter-specific Ea+Ef pairs (P → g1); their offspring of the first generation (developed from cocoons deposited over 3–4 months and separated soon after hatching) were genotyped as Ea, Ef, or hybrids (H). Genotyped adult virgin hybrids of the first generation (H^g1^) were paired with virgin specimens of Ea or Ef (backcrossed) or with another virgin hybrid, forming the pairs (H^g1^+Ea), (H^g1^+Ef), (H^g1^+H^g1^), respectively (g1 → g2). The offspring from cocoons produced over a three-four month period was genotyped. Next, the virgin hybrids of the second generation H^g2^ were joined in pairs with virgin specimens of parental species (backcrossed) or with other hybrids, i.e. forming (H^g2^+Ea), (H^g2^+Ef), (H^g2^+H^g2^) pairs, respectively (g2 → g3). If viable progeny was produced, then the procedure was repeated further to obtain the fourth generation (g3 → g4) (Table 1).

**Table 1:** Scheme of experiments on hybrid existence and hybrid fertility. *Animals generated as described in [11].

Generations	parents	offspring expected		
P → g1	Ea+Ef: L/L, P/L, L/K, L/L*	Ea	Ef	H^g1^
g1 → g2	H^g1^+Ea/Ef/H^g1^	Ea	Ef	H^g2^
g2 → g3	H^g2^+Ea/Ef/H^g2^	Ea	Ef	H^g3^
g3 → g4	H^g3^+………	Ea	Ef	H^g4^

## Results

### Lack of reproduction of isolated virgin Ea and Ef specimens and induction of copulatory behavior by partners from closely related earthworms

Among over 300 earthworms from present studies, only a few sterile cocoons appeared in soil with Ea, Ef or hybrids from L, P, and K populations kept in isolation from hatching for at least 14 weeks. Copulation and extensive cocoon production started soon after joining with closely related Ea or Ef earthworms or their hybrids.

### *Dendrobaena veneta* (Dv) did not induce copulatory behavior of Ea or Ef

Cocoons were not found over a 14 week period in boxes with pairs of freshly hatched specimens of Ea or Ef and *Dendrobaena veneta* (Dv), i.e. in three boxes with Ea+Dv pairs, and three boxes with Ef+Dv pairs; cocoons appeared soon after changing partners to three conspecific Dv+Dv pairs, and three interspecific Ea+Ef pairs. Three months later, the majority of 30 cocoons from the conspecific Dv+Dv groups were fertile giving 20 hatchlings, while most of 30 cocoons from the interspecific Ea+Ef groups were sterile as they gave two hatchlings only.

### First generation offspring of Ea+Ef pairs derived from French (L), Hungarian (P) and Polish (K) laboratory stocks

Adult specimens of virgin Ea and Ef earthworms from L, P, and K populations joined in Ea+Ef pairs started extensive cocoon production that was frequent in all boxes with L/L, P/L, and L/K pairs. Despite the presence of cocoons laid over a three-four month period after pairing, some boxes did not contain hatchlings, either due to sterility of some cocoons and/or early death of some embryos/hatchlings, as a few very small short-lived hatchlings were observed. The surviving hatchlings were cultured until maturation and 37 of them were genotyped (Table 2). Among offspring of the five L/L pairs (both partners from L) there were four pure Ea (aAA.L/L) specimens, three Ef (fFF.L/L) earthworms, and only one Ea-derived hybrid (aAF.L/L) earthworm. The latter developed from a maternal ‘aA’ ovum bearing mitochondrial DNA and one complement of the nuclear Ea genome that had been fertilized by Ef sperm contributing the nuclear ‘F’ paternal genome (origin explained in inset of Fig 1). Among 18 genotyped offspring of nine out of 11 P/L pairs (aAA.P/P+fFF.L/L), three Ea (aAA.P/P) individuals and fifteen aAF.P/L hybrids appeared, the latter from ova of Pecs-derived maternal specimens (P–ovum as the first letter) fertilized by sperm from an Ef1.L-derived earthworm (L–sperm as the second letter); Ef1 earthworms were not obtained from these crosses. Among 11 genotyped offspring of six out of seven L/K pairs (aAA.L/L+f2FF.K/K) nine Ef specimens f2FF.K/K and only two hybrids aAF.L/K were obtained; Ea earthworms were not recovered from these crosses (Table 2 and inset of Fig 1). In the L/L* group, twenty hybrids derived from aAA.L/L+f2FF.L/L pairs (Podolak et al., 2020) were used for further studies.

**Table 2:** First generation offspring of laboratory paired Ea+Ef earthworms derived from Lille (L, France), Pecs (P, Hungary) and Kluczbork (K, Poland) forming L/L, P/L, L/K groups.

Groups	Ea+Ef populations	pair numbers with offspring/total	Numbers of genotyped offspring		
			Ea	Ef	Hybrids H^g1^ L/L, P/L, L/K groups (first letter for Ea, second for Ef)
L/L	aAA.L/L+fFF.L/L	5/5	4 aAA.L/L	3 fFF.L/L	1 **aAF.L/L**
P/L	aAA.P/P+fFF.L/L	9/11	3 aAA.P/P	0	15 **aAF.P/L**
L/K	aAA.L/L+f2FF.K/K	6/7	0	9 f2FF.K/K	2 **aAF.L/K**
L/L*	aAA.L/L+f2FF.L/L	6/7	0	0	*20/67 **aAF.L/L**

*Experiments performed by [11].

All new DNA sequences obtained in present study were added to the GenBank (accession numbers: MT090081-MT090135, MT133042-MT133241 and MT126828-MT126996). The COI-gene based phylogram of genotyped parental specimens and first generation offspring of interspecific pairs formed distinct Ea and Ef clades, the latter with two distinct lineages, Ef1 and Ef2 (Fig 1).

On the Ea branch of the COI-based phylogram, there are 18 inter-specific aAF hybrids from Ea-derived ‘aA’ ova cross-fertilized by Ef-derived ‘F’ spermatozoa, fifteen of them from P/L (aAF.P/L), one from L/L (aAF.L/L), and two from L/K (aAF.L/K) groups, and seven ‘pure’ Ea specimens (aAA), four of them from Lille (aAA.L/L) and three from Pecs (aAA.P/P). The Ef branch contains three fFF.L/L (Ef1.L) earthworms from the French group and nine f2FF.K/K (Ef2.K) pure Ef species from the Kluczbork groups. ‘Pure’ Ea or Ef specimens derived from ‘aA’ or ‘f2F’ova self-fertilized by ‘A’ or ‘F’ spermatozoa, respectively. The hypothetically possible fFA hybrids derived from Ef-ova fertilized by Ea spermatozoa were absent (compare inset and body of Fig 1).

### Progeny of interspecific P/L pairs of earthworms; fertility of first and second generation hybrids from various populations

**Fig 2:**
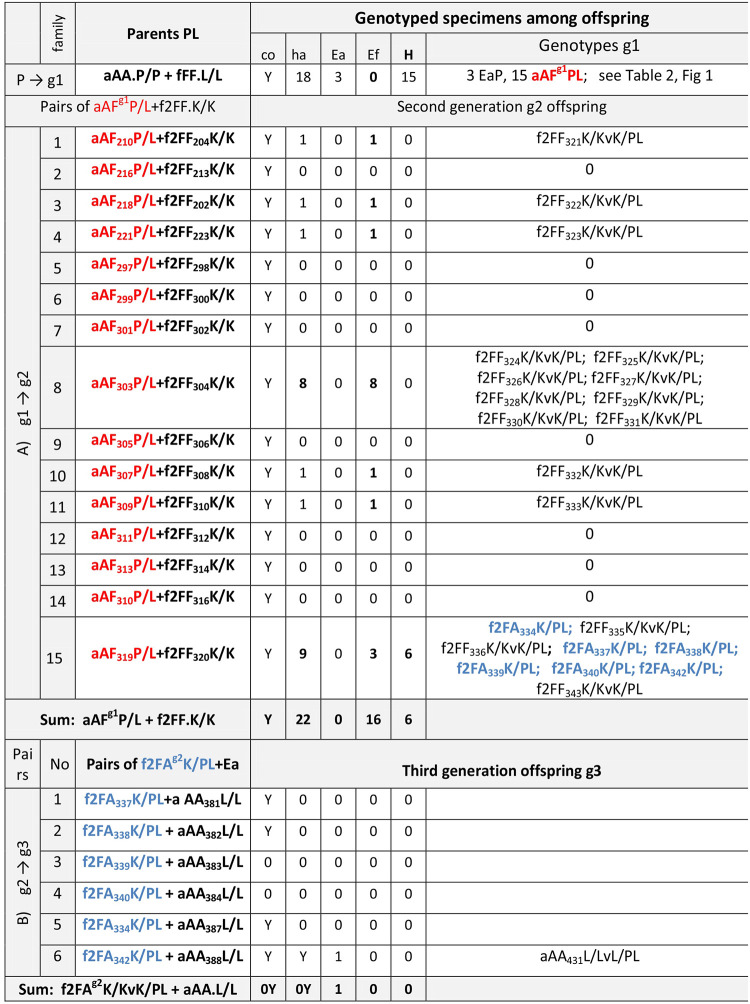
Fertility of backcrosses of the first and second generation EaxEf hybrids from three different sources. Pecs (P, Hungary); Lille (L, France), and Kluczbork (K, Poland). Cocoons (co) and hatchlings (hat), Ea, Ef, and hybrids (H) were present (Y) or absent (0). Hybrids Ea-derived aAF are marked in red and Ef-derived f2FF in blue.

**Fig 3:**
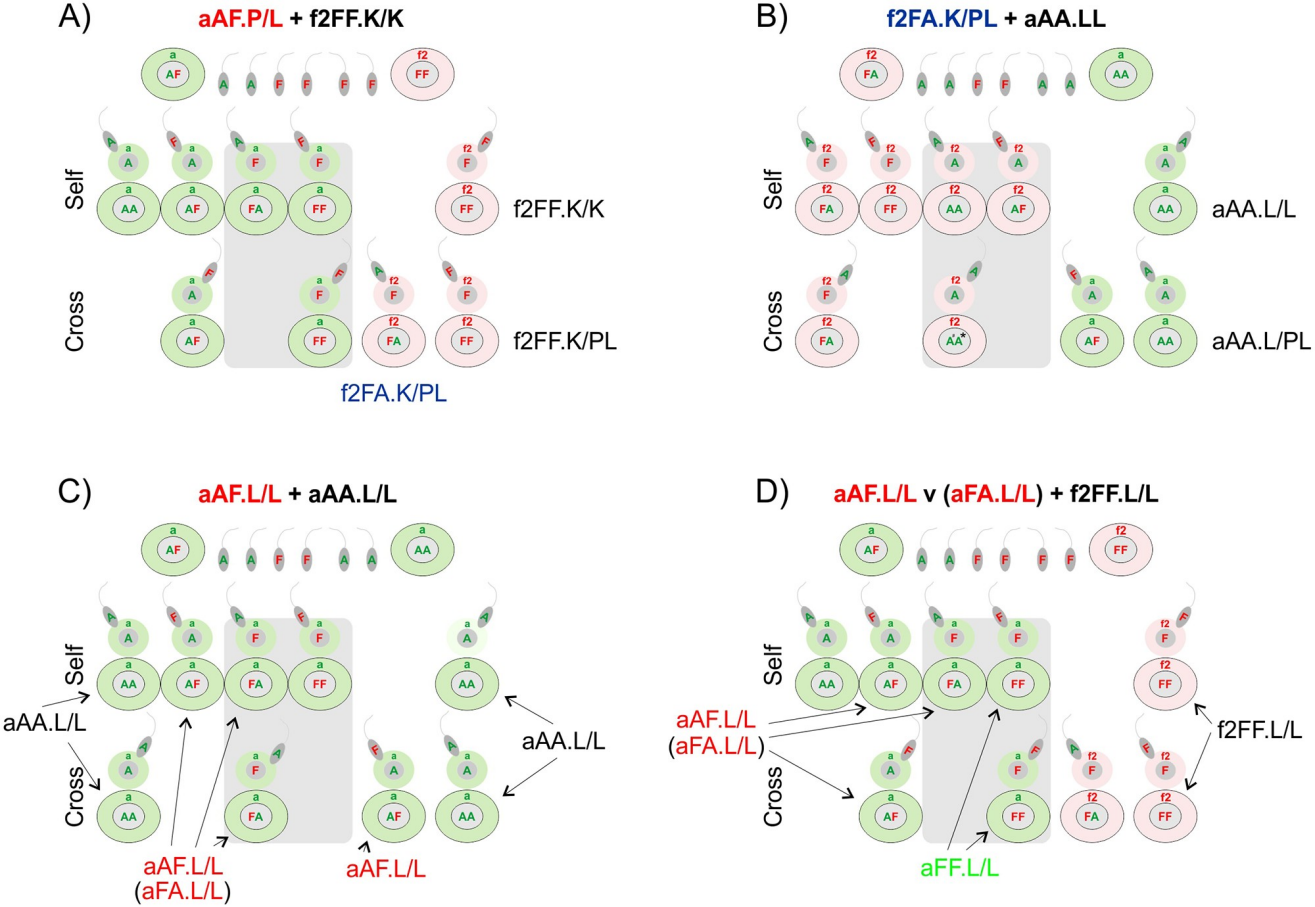
Schemes of mating of inter-specific hybrids with the parental Ea or Ef specimens from various populations (L, P, K). Schemes of cells of parental earthworms, their gametes (ova, spermatozoa) and zygotes; shading indicates mitochondrial-nuclear conflicts. Symbols are the same as in Fig 1.

Fifteen specimens of first generation aAF.P/L hybrids, being the offspring of parents from the P/L (Hungary-France) group (Table 2) were mated with 15 f2FF.K/K from Poland (g1 → g2, Fig 2A). All pairs produced cocoons, but hatchlings developed only in seven of them. Pair number 15 (aAF.P/L+f2FF.K/K) gave six f2FA hybrids derived from f2F ova of f2FF.K/K earthworms cross-fertilized by the ‘hybrid’ PL spermatozoa (potentially containing recombinated material from P and L earthworms) of aAF.P/L hybrid partner, thus marked as f2FA.K/PL, accompanied by three ‘pure’ Ef2 specimens, the latter being either self-fertilized (f2FF.K/K) or cross-fertilized by hybrid-derived PL spermatozoa (f2FF.K/PL). Because in the present experiments it is impossible to distinguish between self-fertilization and cross-fertilization in f2FF specimens, they are marked as f2FF.K/KvK/PL (v stands for versus) to emphasize both possibilities. The remaining f2FF specimens among offspring of all hybrid-Ef2 pairs may also have originated as a result of either self- or cross-fertilization (Figs 2A and 3A).

The f2FA.K/PL second generation hybrids were paired with EaL (aAA.L/L) specimens (g2 → g3, Fig 2B). Cocoons appeared in four out of six pairs, and only one hatchling developed from the Ea-derived cocoon of pair No. 6. This specimen was genotyped as aAA, i.e. derived from Ea ovum either self-fertilized (aAA.L/L) or cross-fertilized by the f2FA hybrid-derived ‘A’-bearing spermatozoon (aAA.L/LvL/PL) (Figs 2B and 3B).

### Progeny of interspecific L/L pairs of earthworms; fertility of hybrids of the first, second and third generation

**Fig 4:**
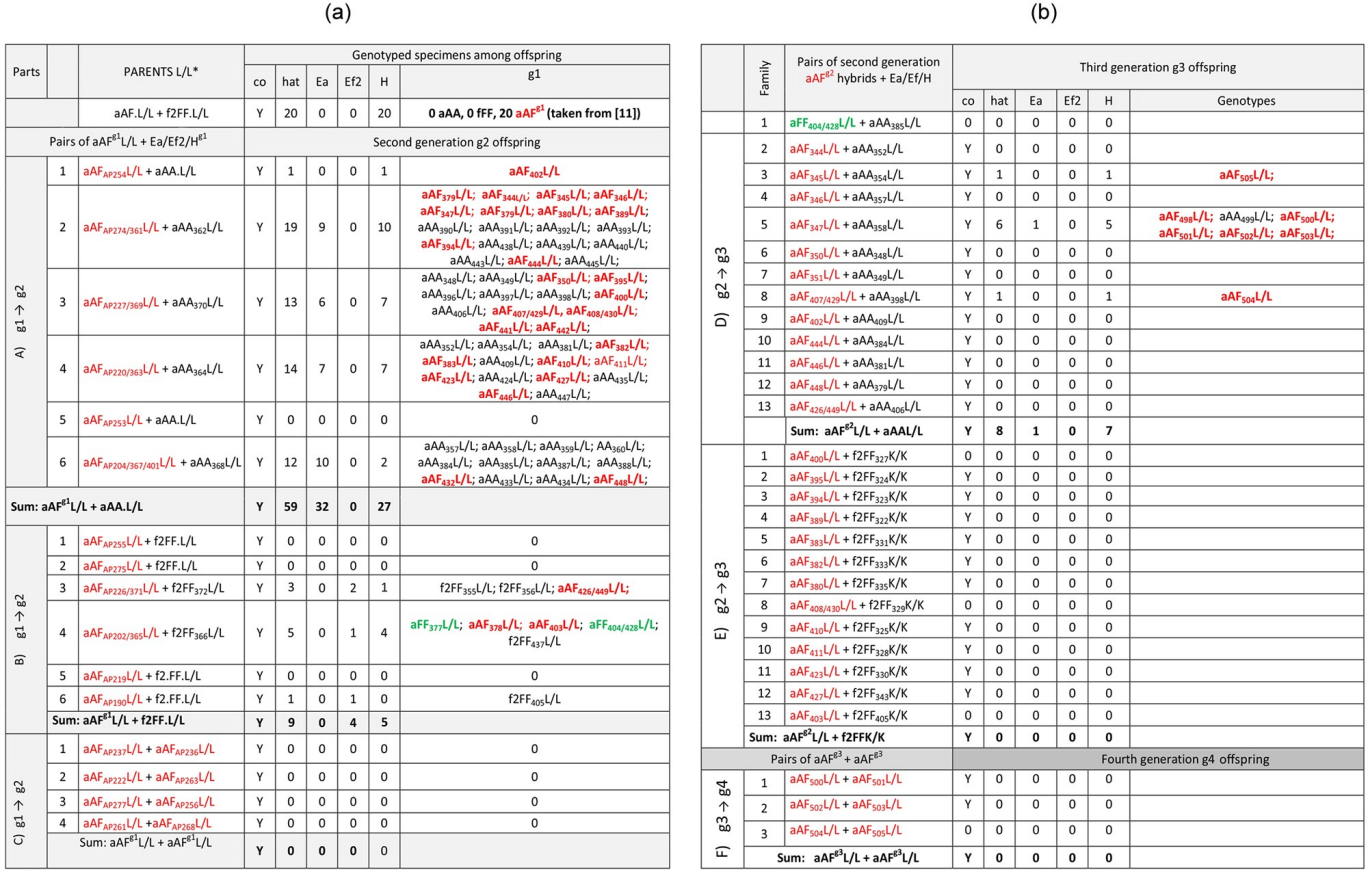
Genotyped progeny of EaL+Ef2L earthworms; fertility of crosses of first, second (Fig 4a) and third generation (Fig 4b) hybrids with parental species or other hybrids. Cocoons (co) and hatchlings (hat) were present (Y) or absent (0). Ea-derived aAF hybrids are marked in red and those with mito-nuclear incompatibility aFF in green. L means earthworms from France and K from Poland.

Twenty specimens of first generation aAF.L/L hybrids derived from aAA.L/L+f2FF.L/L pairs from the L/L* group (Table 2, [11]) were backcrossed with partners from Lille (Fig 4a and 4b), either Ea (6 pairs) or Ef2 (6 pairs), or with other aAF.L/L hybrids (4 pairs) (g1 → g2, Fig 4a, parts A,B,C, respectively). Cocoons were present in all boxes, but hatchlings were present in all but one H^g1^+Ea pairs (Fig 4aA), in three of six H^g1^+Ef pairs (Fig 4aB), and in none of four H^g1^+H^g1^ pairs (Fig 4aC).

Among 59 specimens of second generation offspring of aAF H^g1^+Ea pairs, there were 27 aAF H^g2^, 32 Ea, whereas Ef earthworms were absent (Fig 4aA). The aAF second generation hybrids could have arisen through four different crosses: 1) cross-fertilization of the Ea ova by hybrid-derived ‘F’ spermatozoa; 2) self-fertilization of the hybrid ‘aA’ ova by endogenous ‘F’ spermatozoa, and also (3 and 4) self- or cross-fertilization of the ‘aF’ hybrid’s ova with a mitonuclear mismatch by the hybrid- or Ea-derived ‘A’ spermatozoa (Fig 3C). The aAF and aFA genotypes are indistinguishable by the use of the nuclear marker analyzed in this work. New generations of the Ea specimens could have emerged through four crosses: either by self- or cross-fertilization of the ‘aA’ ova of either ‘pure’ Ea or aAF hybrid-derived sperm (Fig 3C).

Among nine specimens of second generation offspring of aAF (H^g1^)+Ef2 pairs (Fig 4aB), we did not find Ea earthworms and recovered only four Ef2 individuals (from the Ef2 ovum either self- or cross-fertilized) (Fig 3D) and five hybrids of the second generation (H^g2^). Three of the latter were aAF/aFA hybrids evidently derived from ‘aA’ or ‘aF’ hybrid ova being either self- or cross-fertilized by ‘F’ or ‘A’ spermatozoa, respectively, giving aAF or aFA specimens (indistinguishable using our methods) (Fig 3D).

The other two were the aAF-derived aFF hybrids with mitonuclear mismatch, i.e. with Ea-specific ‘a’ mitochondrial genomes from the maternal aAF H^g1^ hybrid and two nuclear ‘FF’ markers. The aFF specimens might have been derived from the ‘aF’ ova of the aAF hybrid either self- or cross-fertilized by the ‘F’ spermatozoa of either Ef or hybrid origin (Fig 3D). This is the first time aFF hybrids occurred in our crossing facilities, but both of them were short-lived. The first one, i.e. specimen aFF_377_L/L, died soon after genotyping, while the second one (aFF_404/428_L/L) died three months after pairing with aAA_385_L/L (Fig 4aB and 4bD).

The only one aFF.L/L (the mentioned above aFF_404/428_L/L individual) and twelve aAF.L/L hybrids from the second generation (H^g2^) were paired with Ea.LL (Fig 4bD). All pairs but ‘atypical’ one (aFF+aAA) gave cocoons, but hatchlings appeared only in three of them, i.e. in pairs No 3, 5, and 8, giving only one Ea specimen and seven aAF hybrids of the third generation (H^g3^) (Figs 3C and 4bD). Thirteen aAF hybrids of the second generation (H^g2^) were paired with Ef2 partners from Kluczbork (f2FF.K/K). Most of them (10 of 13) gave cocoons but hatchlings were absent (Fig 4bE). The remaining 6 aAF H^g3^ hybrids were joined in three H^g3^+H^g3^ pairs; two of them gave cocoons but none hatchlings (Fig 4bF).

### Summary of the main results on hybridization

**Fig 5:**
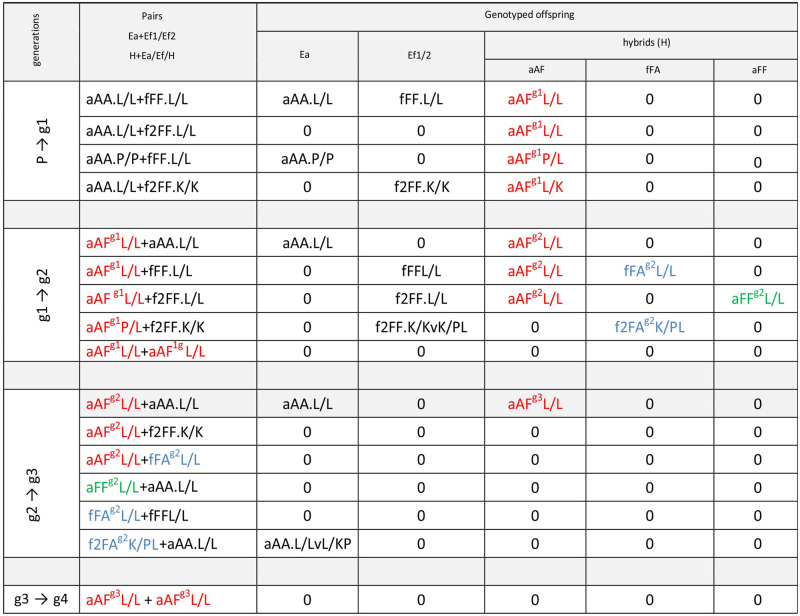
Fertility of hybrids. Hybrid offspring of interspecific pairs of Ea+Ef1/Ef2 (P→g1) from France (Lille, L), Hungary (Pecs, P) and Poland (Kluczbork, K) and of subsequent generations of their hybrids (H): g1 → g2; g2 → g3; g3 → g4 demonstrated lowered fertility with each subsequent generation. Ea—aAA; Ef1—fFF; Ef2—f2FF; H—aAF; fFA; aFF.

During independent experimental series ([9–11]; present investigations), fertile Ea-derived hybrids but no Ef-derived hybrids occurred among first generation offspring of Ea+Ef1 and Ea+Ef2 pairs from French, Hungarian, and Polish laboratory stocks (P → g1), sometimes accompanied by Ea and/or Ef specimens resulting from self-fertilization. The first generation of Ea-derived hybrids backcrossed with Ea gave second generation Ea-derived hybrids sometimes accompanied by Ea specimens, while backcrossed with Ef gave a new generation of Ea-derived hybrids, and very rare sterile aFF hybrids with mito-nuclear mismatch, and also Ef1-derived or Ef2-derived hybrids, sometimes accompanied by Ef1 or Ef2 specimens (g1 → g2). Ea-derived hybrids of the second generation mated with Ea gave rare specimens of the third generation: Ea-derived hybrids and Ea earthworms, while Ef-derived hybrids paired with Ea gave only one Ea specimen and no hybrids (g2 → g3). Pairs of hybrids in g1 → g2, g2 → g3 and g3 → g4 consistently gave no offspring even if cocoons were produced (Fig 5).

## Discussion

Reproductive isolation between Spanish and Brazilian specimens of *Eisenia andrei* and *E*. *fetida* [5] runs counter to the incomplete isolation of these species from French, Hungarian and Polish laboratory stocks ([9–11]; present results). In the latter case, the first generation offspring of Ea+Ef pairs included Ea-derived hybrids that developed from Ea-ova fertilized by Ef-spermatozoa, sometimes accompanied by pure Ea and/or Ef specimens but not by Ef-derived hybrids. First generation Ea-hybrids backcrossed with Ea produced some ‘pure’ Ea specimens and second generation Ea-hybrids. The latter backcrossed again with Ea gave only a few ‘pure’ Ea and Ea-hybrids of the third generation, thus the reproductive capability of Ea-derived hybrids was gradually impaired. Such asymmetric hybridization and depressed reproductive success was also described in snails [16–18].

Subsequent generations of aAF hybrids may appear in some backcrosses of Ea-derived hybrids with the Ea or Ef partners, accompanied by some sterile Ef-derived (fFA) hybrids from Ef-ova fertilized by hybrid-derived ‘A’ spermatozoa, and a few unique aFF hybrids. The asymmetric hybridization between Ea and Ef indicates that the Ea ova accept the Ef spermatozoa and the resulting embryos develop relatively easily, while successful hybridization in the opposite direction is very rare. This observation may be connected with higher fecundity of Ea than Ef [e.g. 11, 19], however the underlying molecular mechanism remains unknown.

The unique sterile and short living aFF specimens among offspring of the aAF+Ef pair were derived from the hybrid’s ‘aF’ ova fertilized within the hybrid’s cocoon either by a endogenous ‘F’ spermatozoon or by the Ef partner’s sperm. The existence of such specimens was considered previously as theoretically possible but of low probability due to mito-nuclear incompatibility resulting in decreased viability of incompatible ‘aF’ ova or early aFF embryos [9]. The present results support this hypothesis, as aFF specimens were extremely rare. Mito-nuclear incompatibility reduces viability [20], but our results suggest that this barrier might be incomplete. It can be affected by some environmental factors as reported in *Drosophila* sp. [e.g. 21, 22]. Incompatibility of mitochondrial haplotype with clusters of nuclear genes was detected in field populations of *Allolobophora chlorotica* and was explained by multiple generations of unidirectional hybridization in nature [23].

The presence of the Ea and Ef specimens among first generation offspring of Ea+Ef pairs is necessarily caused by self-fertilization of parental individuals, perhaps due to admixture of endogenous sperm with that of the partner’s during prolonged copulation followed by storage of mixed spermatozoa in spermathecas, resulting in facilitated self-fertilization. We cannot exclude that the aAA or fFF genotypes among offspring of hybrids might be achieved by DNA recombination within aAF or fFA specimens. However, the presence of ‘pure’ Ea and Ef specimens among offspring of backcrosses of hybrids with one of the parental species may be the result of either self-fertilization by endogenous spermatozoa (facilitated self-fertilization, as in the first generation offspring of Ea+Ef pairs) or cross-fertilization by hybrids’ spermatozoa; moreover, the pure specimens may also originate by hybrid self-fertilization. Paternity might be conclusively established by analysis of a wide array of species-specific DNA sequences like microsatellites [e.g. 24]. In the case of cross-fertilization, both in the first and next generations, some species-specific (or population-specific) genes might be transferred to the genome of the partner. Such processes are responsible for introgression of some genes from Ea through Ea-derived hybrids to Ef. This was exemplified by the transfer of unidentified genes responsible for Ea-specific fluorescence [25, 26] through Ea-derived hybrids to some Ef specimens, and their further dispersal within Ef [10].

In contrast to the uniparental reproduction of some Ea and Ef specimens from Spain [12], the French, Hungarian, and Polish Ea/Ef earthworms produced only a few sterile cocoons when kept in isolation after hatching [11] or no cocoons when maintained from hatching with highly divergent species such as *Dendrobaena veneta*. Cocoon production and reproduction was induced by recognition of a partner from the same genus, like Ea, Ef, or their hybrids. Presumably, reproductive activity might be induced by sensing a specific blend of chemical signals covering the body surface of congeners, but not those of distantly related earthworms possessing a different mixture of coelomic fluid and/or body-covering compounds, as exemplified by *Dendrobaena* sp. versus *Eisenia sp*. [13, 14]. The recognition of semiochemicals of the related partner might be mediated by chemoreceptors and/or the sensory tubercle on the body [27, 28] and might stimulate copulatory behavior controlled by factors derived from the neuroendocrine network, well-developed in lumbricid species [e.g. 15, 29–34], which in turn might prompt cocoon production and reproduction.

In summary, reproductive isolation between Ea and Ef from France, Hungary and Poland is incomplete and asymmetric; Ea-derived hybrids are fertile, but their reproductive capabilities are depressed in subsequent generations; reproduction is absent in isolated virgin earthworms, but self-fertilization may be induced by contact with closely related Ea, Ef, or hybrid partners. In contrast, Ea and Ef from Spanish and Brazilian sources are reproductively fully isolated [5], while virgin specimens are capable of reproduction in isolation [12]. These striking differences make earthworms from various localities and/or various laboratory stocks attractive models for studies on mechanisms of speciation by analysis of more informative genetic markers (such as a panel of genome-wide single nucleotide polymorphisms). Presumably, species boundaries are already better established in Ea and Ef specimens from Spanish laboratories than in those from Krakow, therefore the former earthworms reproduced exclusively within their own species, while the latter were more likely to copulate and give fertile progeny with any similar partner. One possible explanation and avenue of further research could involve the influence of climatic instability on reproductive strategy and life history in earthworms.
